# A Nationwide Network to Provide Supervised Exercise Therapy and Lifestyle Counseling for All Patients with Non-Communicable Diseases: Chronic CareNet

**DOI:** 10.3390/ijerph17165999

**Published:** 2020-08-18

**Authors:** Anneroos Sinnige, Maik Sliepen, Marc R. Scheltinga, Joep A.W. Teijink

**Affiliations:** 1 Department of Vascular Surgery, Catharina Hospital, 5623 Eindhoven, The Netherlands; anneroos.sinnige@catharinaziekenhuis.nl; 2 CAPHRI Research School, Maastricht University, 6211 Maastricht, The Netherlands; 3 Chronic CareNet, 5623 Eindhoven, The Netherlands; maik.sliepen@catharinaziekenhuis.nl; 4 Department of Surgery, Máxima Medical Center, 5631 Eindhoven/Veldhoven, The Netherlands; M.Scheltinga@mmc.nl

**Keywords:** non-communicable diseases, physical therapy, supervised exercise therapy, integrated care network

## Abstract

Non-communicable diseases (NCDs) are a major cause of disability and mortality worldwide. Physical inactivity is an important contributor to the development of NCDs. Increasing physical activity through supervised exercise therapy (SET) is proven to be effective, and is a key component in both the prevention and treatment of most NCDs. However, only a minority of patients with NCDs receive this treatment, mainly due to an insufficient number and poor accessibility of specialized physical therapists. The aim of this article is to describe a solution that, if indicated, enables all patients with NCDs in the Netherlands to receive SET by a specialized physical therapist: Chronic CareNet. Chronic CareNet is a nationwide network of specialized physical therapists, providing high quality SET and lifestyle counselling to patients with NCDs, initially focusing on peripheral arterial disease, chronic obstructive pulmonary disease and coronary heart disease. The network evolved from ClaudicatioNet. In order to monitor quality of care, therapists enroll in a continuous educational program, and process and outcome indicators are collected by all therapists, which can be compared with a nationwide benchmark (quality system). A robust infrastructure of information and communication technology provides an online care finder and referral system to locate and refer to nearby therapists. All elements of Chronic CareNet are essential, to ensure that all patients in the Netherlands have access to a nearby specialized therapist.

## 1. Introduction

Non-communicable diseases (NCDs) are a major cause of chronic disability and responsible for 71% of all deaths worldwide. Cardiovascular disease (CVD) is the most prevalent type, and accounts for almost half of all NCD-related deaths [1]. Other common NCDs are diabetes mellitus, cancer and chronic obstructive pulmonary disease (COPD). Over the past decades, NCD-related death rates have risen substantially, due to population growth and aging, and are expected to continue to rise [1,2].

People develop NCDs due to a combination of genetic, physiological, environmental and behavioral factors. Behavioral factors have the largest impact on developing NCDs, and are responsible for approximately half of the NCD-related deaths [3]. The four major behavioral factors are tobacco use, excessive alcohol consumption, unhealthy diet and physical inactivity [1]. As these risk factors are all modifiable, they are an important target for both the prevention and treatment of NCDs [1].

Physical inactivity alone is responsible for 6–10% of all NCD-related deaths worldwide [1,4,5]. Adopting a healthy behavioral pattern by becoming more physically active is found to substantially reduce disability and risk of morbidity and mortality associated with NCDs [6,7,8]. However, physical activity of most patients with NCDs is insufficient. Consequently, the World Health Organization (WHO) stated that an increase in physical activity, defined as ‘any bodily movement produced by skeletal muscles that requires energy expenditure’, is an important tool for reducing the growing global burden of NCDs [9].

Exercise is a strategy to increase physical activity and is defined as planned, structured and repetitious physical activity aimed to explicitly improve physical fitness [9]. Structured exercise therapy (ET) is recommended in prevailing disease specific guidelines for a variety of NCDs, including peripheral arterial disease (PAD), coronary heart disease, COPD and diabetes [10,11,12,13,14,15,16,17,18,19]. ET is effective in the prevention and treatment of these NCDs, as physical performance capacity and muscle strength increase, ultimately leading to improved self-sustainability and daily physical activity [19,20,21]. In addition, ET beneficially affects other NCD-related factors, such as high blood pressure, high body fat mass, chronic inflammation, atherosclerosis and insulin resistance [19,21,22]. It also safely increases heart function in patients with NCDs [23].

Even though the effectiveness and safety of ET for patients with NCD is undisputed, adhering to physical activity recommendations remains a major challenge for patients [1,24]. Studies have shown improved treatment outcomes and better long-term adherence to physical activity recommendations when patients are supervised by a specialized physical therapist (supervised exercise therapy; SET) compared to other training modalities (e.g., home-based ET). Therefore, all patients with NCDs for whom ET is indicated according to the guidelines should ideally receive SET by a specialized therapist, in order to achieve the best treatment outcomes and adherence to physical activity [25,26,27].

Despite the scientific evidence and guidelines, only a small minority of patients with NCDs are actually referred for SET: less than 5% of patients with newly diagnosed COPD and 11.7% of the patients indicated for heart rehabilitation receive rehabilitation programs, as recommended by the guidelines [28]. This can mainly be ascribed to the availability and accessibility of specialized therapists by referring health professionals (e.g., GPs, vascular surgeons, lung specialists, cardiologists).

The aim of this article is to describe how a Dutch approach called ‘Chronic CareNet’ (Chronisch ZorgNet in Dutch) deals with persistent obstacles, in order to enable all patients with NCDs in the Netherlands to receive SET and lifestyle counselling by a specialized physical therapist, as indicated by prevailing guidelines.

## 2. ClaudicatioNet, a Dutch Network and Start of Supervised Exercise Therapy for All Patients with PAD in the Netherlands

At the start of this millennium, ET was recommended as primary treatment for patients with PAD. However, follow-up of this recommendation was incidental as only fourteen percent of all patients diagnosed with PAD actually received SET in 2009 [29].

In 2010, The ExitPAD triall by Nicolai et al., [30] showed that SET was more effective than walking advice to improve walking distance and quality of life in patients with PAD. Based on the results of this multicenter randomized controlled trial, ClaudicatioNet was initiated in 2011 in the Netherlands to provide standardized high-quality SET in combination with lifestyle counseling for patients with one specific NCD: PAD. The addition of lifestyle counseling to SET is preferable to further optimize treatment results, as physical activity is only one aspect of an healthy lifestyle. Over the past nine years, this foundation has grown into a nationwide network, with over 2000 specialized physical therapists, realizing SET and lifestyle counseling that is accessible for all patients with PAD in the Netherlands. Specialization includes a baseline training, motivational interviewing training, disease specific courses (for example smoking cessation and fall prevention training) and updating knowledge through attendance of conferences.

ClaudicatioNet has demonstrated its added value as a network for high-quality and cost-effective care for patients with PAD. For instance, ClaudicatioNet studied safety and cost-effectivity of PAD, resulting in better reimbursement for patients and the uptake of the stepped care approach (SET as primary treatment) in guidelines [29,31]. Furthermore, specialized therapists for SET and lifestyle counseling are available with nationwide coverage and these therapists can be easily found and referred to via an online care finder and referral system. Addressing the obstacles for patients and referring health professionals resulted in highly improved adherence to the stepped care approach, as shown by Dutch health insurance claims data. In 2017, 87% of all patients diagnosed with PAD primarily received SET and lifestyle counseling. The same health insurance claims data also showed that after five years of the initiation of SET, 83% of all patients with PAD is still free of any vascular invasive intervention [32], thereby confirming the effectiveness of SET. Additionally, adherence to the stepped care approach can result in saving up to EUR 33.0 million for the Dutch population, annually [29].

## 3. SET and Lifestyle Counselling for All Patients with NCDs: Chronic CareNet (Chronisch ZorgNet)

As described above, in the past years, most obstacles for guideline adherence for patients with PAD were removed. Consequently, most patients with PAD currently receive SET. However, the majority of patients with other NCDs are still not referred for SET and lifestyle counseling. Two important obstacles remain for patients with NCDs (other than PAD): poor accessibility of specialized therapists for referring health professionals and an insufficient number of available specialized physical therapists.

Although some adequate regional initiatives are available in the Netherlands, no national coverage of specialized physical therapy treatment for patients with NCDs exists. To make SET available for all patients with NCDs (in accordance with guideline recommendations), the solid base, experience and successful aspects of ClaudicatioNet have been expanded to Chronic CareNet, starting early 2020. The goal of Chronic CareNet is to create a nationwide network of specialized physical therapists who provide high quality and transparent SET with lifestyle counseling for all patients with NCDs. The network aims to provide ‘the right care in the right place’, meaning that all patients with NCDs for whom therapy is indicated should receive this. Chronic CareNet created a specialization program, initially for PAD, COPD and coronary heart disease and expanded the extensive infrastructure of information and communication technology (ICT) and regional networks of ClaudicatioNet.

### 3.1. Specialized Physical Therapists

The treatment of patients with NCDs can be complex, as a substantial portion of these patients suffers from multiple chronic diseases and comorbidities simultaneously [33]. Currently, every physical therapist—whether specialized in the treatment of NCDs, or not—can treat this patient category: a recipe for suboptimal results. Although specialization programs are available for physical therapists, it is still generally accepted for therapists to treat a broad range of conditions. We believe that, in order to attain the best possible results, differentiation in the physical therapy profession is necessary. Quality of patient care will benefit to a large extent, if only therapists specialized in NCD-specific exercise therapy and lifestyle counseling treat these patients.

Chronic CareNet physical therapists have to meet certain network quality criteria to participate in the network, including baseline training on evidence-based practice for treating patients with NCDs and annual attendance of relevant training. Specialization in multiple NCDs is beneficial, since disease specific exercise programs share the aim to reduce symptoms and improve self-sustainability by increasing physical capacity. Additionally, the different NCDs commonly have the same preventable risk factors, meaning comparable lifestyle modifications are important components of the treatment (irrespective of the disease) [32]. Although the overlap in exercise recommendation and lifestyle modifications can act as a good base for therapy, the therapy should be adapted to disease and patient specific needs. Chronic CareNet therapists are trained to execute personalized exercise training, lifestyle counseling, monitor medication adherence, collaborate with other health professionals and, if needed, react adequately to disease specific alarm symptoms during the treatment. A sufficient number of specialized physical therapists would be achieved when every patient with NCDs has a therapists available nearby their residential address.

### 3.2. ICT Infrastructure

In order to provide transparency on the availability of specialized therapists and to monitor the quality of care, an extensive ICT infrastructure is implemented in Chronic CareNet. This infrastructure includes: (1) an online carefinder and referral system; (2) a personal account for each participating therapist; and (3) a quality system, in which routinely measured data are collected and visualized.

#### 3.2.1. Online Carefinder and Referral System

One of the major challenges in adhering to guideline recommendations on SET is the availability and accessibility of specialized therapists. To find these therapists in the proximity of the residential address of the patient, an online carefinder (https://chronischzorgnet.nl/nl/zorgzoeker) is available to search for specialized physical therapists (PAD, COPD, cardiac rehabilitation) based on postal code. Furthermore, an online referral system can be used by health care providers (physicians, nurse practitioners and GP-assistants) to easily refer a patient. The therapist receives contact information of the newly referred patient in his/her personal account, and initiates contact with the patient within three days. This system was implemented as a solution for drop-outs after referral and delays in treatment start. If the therapist fails to contact the patient within 3 days, the next nearby therapist is automatically instructed to contact the patient, thereby guaranteeing a timely start of therapy. Chronic CareNet referral data show that on average, the treatment is initiated within 3.2 days after referral.

#### 3.2.2. Personal Therapist Account

Every participating physical therapist has access to a personal account, which contains a portfolio with a complete resume of all specialization and training courses. This resume is publicly available for patients and referring healthcare providers on the carefinder. Chronic CareNet monitors the training activities via the therapist account. Therapists receive newly referred patients and send standardized feedback letters at pre-specified intervals (or more often if necessary) to referring health care professionals via their account.

#### 3.2.3. Chronic CareNet Quality System

In 2015, the ClaudicatioNet quality system was initiated to monitor the quality of treatment for patients with PAD. This quality system is a large database that contains routinely measured treatment outcomes (e.g., walking distance and quality of life) of patients treated by Chronic CareNet physical therapists. Data collection in the quality system is expanded for relevant health indicators of the other NCDs. With these data, the quality of care and variations in therapy outcome can be monitored to detect under- and overperforming therapists to intervene or learn form. Insight into treatment outcome data and benchmark comparisons of regional and national outcomes are provided for individual therapists through their personal account. Furthermore, scientific research is conducted to further improve health care.

### 3.3. Regional Networks

Each Chronic CareNet therapist participates in a regional network. These regional networks enable Chronic CareNet therapists to cooperate more closely with colleagues and other healthcare professionals within their region. Every network organizes meetings to exchange knowledge, make agreements with relevant healthcare professionals of the region and organize regional patient activities. All members of the regional network have access to an online community in their personal accounts, in order to arrange meetings and share information.

## 4. Discussion

Despite scientific evidence and guideline recommendations, only a minority of patients with NCDs in the Netherlands are referred for SET as primary treatment. This low referral rate is mainly due to insufficient and poorly accessible specialized physical therapists. The aim of this article was to discuss how the Dutch approach Chronic CareNet deals with obstacles that hinder the availability to receive SET and lifestyle counselling by a specialized physical therapist for all patients with NCDs in the Netherlands. In the above, it was discussed how the network is organized to realize the availability and accessibility of a sufficient number of specialized therapists for patients and referring health professionals. Although the availability of specialized therapists is essential to realize improvements in patient care, other factors should not be overlooked. We therefore discuss the importance of collaboration with relevant stakeholders and proper reimbursement for the patients.

Healthcare for patients with NCD’s is not only centered around physical therapists. Not only do therapists cooperate with other health professionals, but Chronic CareNet also collaborates with national associations of general physicians and medical specialists. Chronic CareNet aims to raise awareness regarding the guidelines, primary treatment for patients with NCD’s and the existence of the network. The collaboration with relevant patient federations creates awareness amongst patients about the disease and its treatment. Finally, cooperating with the physical therapy professional association (Royal Dutch Society for Physical Therapy) results in the quality system and updated treatment guidelines for patients with PAD.

Besides insufficient and poorly accessible specialized physical therapists, lack of reimbursement might hamper implementation of supervised exercise programs for patients with NCDs. The lack of reimbursement for SET might prevent patients to choose this treatment. This is especially the case for NCD patients, as they often have a low socio-economic status [34]. In the Netherlands, SET for patients with PAD is fully reimbursed since 2017 through basic health insurance, on the condition that the therapy is provided by a Chronic CareNet therapist. Scientific studies have shown that 6% of the healthcare costs per patient with peripheral arterial disease can be saved by primarily prescribing SET compared to invasive treatment [29,31]. Although similar trends regarding cost savings can be expected for COPD and coronary heart diseases, SET is not fully reimbursed for every patient with these diseases in the Netherlands. Patients with COPD are reimbursed up to a certain number of sessions based on their disease burden, which might be inadequate, especially for the less severe COPD gold stages. Cardiac rehabilitation (including SET) for patients with coronary heart disease is only reimbursed if SET is executed in a hospital setting or rehabilitation clinic. The adherence rate of cardiac rehabilitation in the Netherlands is very low, as only 11.7% of patients who are indicated for cardiac rehabilitation actually receive it. One of the main reasons for low adherence is the distance to hospital or rehabilitation clinic [28]. As patients with non-complex coronary heart diseases can be treated outside the hospital setting, the patient can train with a specialized therapist nearby their residential address, overcoming a major obstacle for adherence.

## 5. Conclusions

Chronic CareNet is a nationwide network of specialized physical therapists, providing high quality SET and lifestyle counselling for patients with NCDs. Chronic CareNet aims to provide ‘the right care in the right place’. The network evolved from ClaudicatioNet, which has already demonstrated its added value as network by realizing high-quality, cost-effective and transparent SET and lifestyle counseling for all patients with PAD in the Netherlands. Chronic CareNet therapists are trained in providing SET in combination with lifestyle counseling for patients with (multiple) NCDs. The main benefits of the Chronic CareNet network are the ease of referring a patient to the right therapist, the timely start of high-quality therapy and the monitoring of quality with the use of gathered treatment outcomes through the quality system.
